# Serum galactose-deficient-IgA1 and IgG autoantibodies correlate in patients with IgA nephropathy

**DOI:** 10.1371/journal.pone.0190967

**Published:** 2018-01-11

**Authors:** William J. Placzek, Hiroyuki Yanagawa, Yuko Makita, Matthew B. Renfrow, Bruce A. Julian, Dana V. Rizk, Yusuke Suzuki, Jan Novak, Hitoshi Suzuki

**Affiliations:** 1 Department of Biochemistry and Molecular Genetics, The University of Alabama at Birmingham, Birmingham, Alabama, United States of America; 2 Department of Nephrology, Juntendo University Faculty of Medicine, Tokyo, Japan; 3 Department of Medicine, The University of Alabama at Birmingham, Birmingham, Alabama, United States of America; 4 Department of Microbiology, The University of Alabama at Birmingham, Birmingham, Alabama, United States of America; INSERM1163, FRANCE

## Abstract

IgA nephropathy is an autoimmune disease characterized by IgA1-containing glomerular immune deposits. We previously proposed a multi-hit pathogenesis model in which patients with IgA nephropathy have elevated levels of circulatory IgA1 with some *O*-glycans deficient in galactose (Gd-IgA1, autoantigen). Gd-IgA1 is recognized by anti-glycan IgG and/or IgA autoantibodies, resulting in formation of pathogenic immune complexes. Some of these immune complexes deposit in the kidney, activate mesangial cells, and incite glomerular injury leading to clinical presentation of IgA nephropathy. Several studies have demonstrated that elevated circulatory levels of either Gd-IgA1 or the corresponding autoantibodies predict progressive loss of renal clearance function. In this study we assessed a possible association between serum levels of Gd-IgA1 and IgG or IgA autoantibodies specific for Gd-IgA1 in serum samples from 135 patients with biopsy-proven IgA nephropathy, 76 patients with other renal diseases, and 106 healthy controls. Our analyses revealed a correlation between the concentrations of the autoantigen and the corresponding IgG autoantibodies in sera of patients with IgA nephropathy, but not of disease or healthy controls. Moreover, our data suggest that IgG is the predominant isotype of Gd-IgA1-specific autoantibodies in IgA nephropathy. This work highlights the importance of both initial hits in the pathogenesis of IgA nephropathy.

## Introduction

IgA nephropathy (IgAN) is the most common primary glomerulonephritis in the world and a frequent cause of end-stage renal disease.[1] IgAN is diagnosed by evaluation of renal biopsy specimen and characterized by mesangial IgA deposits as the predominant or codominant immunoglobulin.[2, 3] Of the two human IgA subclasses, IgA1 and IgA2, only IgA1 is found in the glomerular deposits of patients with IgAN.[4, 5] Unlike IgA2, IgA1 has clustered *O*-glycans in the hinge region and the degree of their galactosylation is central to the pathogenesis of IgAN.[6] We have proposed that IgAN is an autoimmune disease with a multi-hit process, wherein IgA1 with some *O*-glycans deficient in galactose (galactose-deficient IgA1; Gd-IgA1) [Hit 1] is recognized by IgG and/or IgA autoantibodies (Gd-IgA1-specific IgG and/or Gd-IgA1-specific IgA, respectively) [Hit 2] to form pathogenic immune complexes [Hit 3].[7, 8] Some of these immune complexes deposit in the kidney, as evidenced by enrichment for Gd-IgA1 glycoforms in the glomerular immune deposits.[9, 10] These Gd-IgA1-containing immune complexes activate mesangial cells and incite glomerular injury [Hit 4].[6] This hypothesis has been supported by multiple studies (for review, see Lai *et al*.[3]).

Prior studies have determined that elevated serum levels of Gd-IgA1 or Gd-IgA1-specific IgG/IgA autoantibodies predict progressive loss of renal clearance function in patients with IgAN.[11, 12] Moreover, the presence of glomerular IgG deposits on kidney biopsy correlate with the extent of mesangial and endocapillary cellularity and associate with poor renal outcome.[13–15] In this study, we sought to assess whether there is an association between serum levels of Gd-IgA1-specific IgG and IgA autoantibodies and their corresponding autoantigen, Gd-IgA1.

## Results

### Serum component analysis

**Fig 1:**
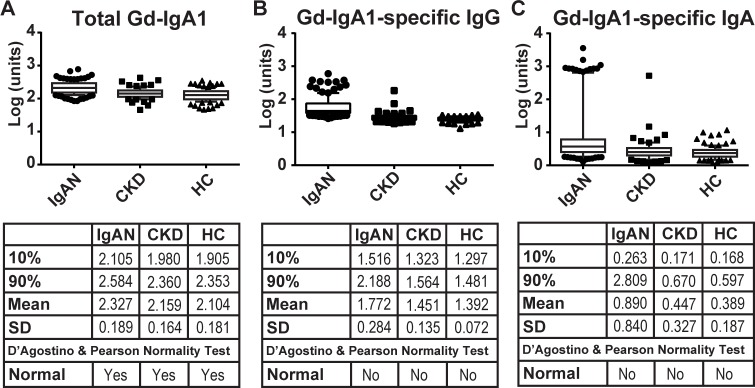
Analysis of total Gd-IgA1, Gd-IgA1-specific IgG, and Gd-IgA1-specific IgA in serum samples from patients with IgAN (IgAN), chronic-kidney disease controls (CKD), and healthy controls (HC). Box and Whisker plots of 10-90^th^ percentiles with additional points shown as circles (IgAN), squares (CKD), and triangles (HC). Group statistics, including the mean and standard deviation (SD) as well as the result of the D’Agostino & Pearson normality tests, are shown in the tables. Log transformation of each measurement is plotted in the corresponding units, as outlined in Methods.

We previously employed ELISA-based assays to determine serum concentrations of total IgA, IgG, Gd-IgA1, Gd-IgA1-specific IgA, and Gd-IgA1-specific IgG in cohorts that included 135 patients with biopsy-proven IgAN, 79 patients with other biopsy-proven chronic kidney diseases (CKD), and 106 healthy controls (HC).[16] Follow-up analysis of the CKD patients identified three individuals with evidence of renal IgA1 deposits and these three individuals were therefore excluded from this study. A description of the CKD cohort is provided in S1 Table. Initial analysis of the levels of the serum constituents found that all exhibited significant skew. In an attempt to normalize the distributions and reduce skew, we performed log transformations of each data set. Log transformation of serum Gd-IgA1 resulted in a normal distribution of values for each cohort. Whereas student t-test analysis of these data identified a significant difference (P<0.0001) between IgAN patients and either CKD or healthy controls, plotting these data highlights the significant overlap between the cohorts (Fig 1A). These findings agree with prior Gd-IgA1 studies that have shown significant overlap of the serum Gd-IgA1 levels in healthy-control and IgAN cohorts.[16, 17]

Log transformation of the values for serum levels of Gd-IgA1-specific IgG or Gd-IgA1-specific IgA did not normalize the distributions of the data (Fig 1B and 1C), as they failed to pass the D’Agostino and Pearson normality test.[18] Using either the raw-data distributions or log transformation of the data, the serum levels of Gd-IgA1-specific IgG in the IgAN and CKD cohorts exhibited a significant positive skew. A similar positive skew was observed for Gd-IgA1-specific IgA in all three cohorts. Of note, the healthy-control cohort exhibited a negative skew for Gd-IgA1-specific IgG. The range of the serum levels for Gd-IgA1-specific IgA or IgG was also significantly tighter for the healthy-control cohort compared to that for either the IgAN patients or CKD patients, as shown by smaller standard deviation (SD) values (Fig 1B and 1C). Mann-Whitney U tests to assess differences of Gd-IgA1-specific IgG between IgAN and CKD cohorts, or either of these two cohorts *vs*. healthy controls, were statistically significant (P<0.0001). Similar significance was observed when comparing Gd-IgA1-specific IgA autoantibodies. Importantly, we observed separation of the healthy-control and IgAN-patient cohorts’ distributions of Gd-IgA1-specific IgG. Notably, the 90^th^ percentile of the Gd-IgA1-specific IgG of the healthy-control cohort (1.481 units) was below the 10^th^ percentile of the Gd-IgA1-specific IgG of the IgAN-patient cohort (1.516 units).

### Dependence on total immunoglobulin levels

We next sought to determine how serum concentrations of the key components of our multi-hit disease model could inherently vary, based on serum concentrations of total IgG or IgA. To address this point, we analyzed the relation between Gd-IgA1-specific IgG or IgA autoantibodies and their respective immunoglobulin pools, as well as Gd-IgA1 and both immunoglobulin pools. Initial analysis found no clear separation of serum total IgG or IgA concentrations (S1A and S1B Fig) among the three cohorts, although the IgAN cohort exhibited elevated serum levels of total IgA compared to both the CKD and healthy-control cohorts (S1B Fig). Neither was a correlation observed between serum concentrations of the autoantibodies (IgG, IgA) and the respective total IgG or total IgA concentrations (S1C and S1D Fig). Analysis of serum concentrations of Gd-IgA1 and total IgG (S1E Fig) found no correlation in any cohort. However, all three cohorts exhibited a correlation between the serum concentrations of Gd-IgA1 and total IgA (S1F Fig).

### Autoantibody dependence on Gd-IgA1

**Fig 2:**
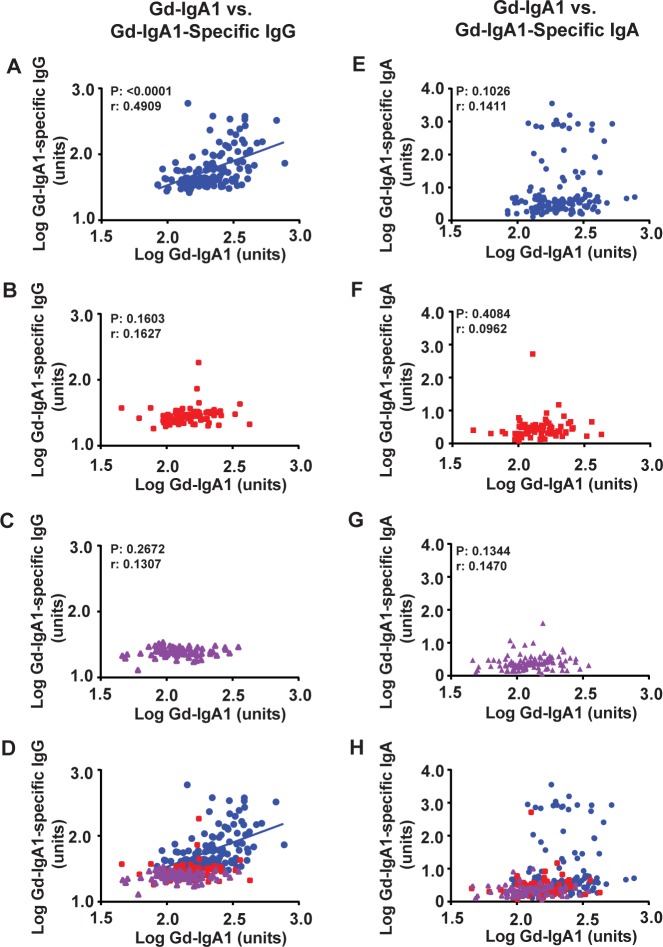
Serum levels of Gd-IgA1-specific IgG autoantibodies correlate with serum levels of Gd-IgA1 only in patients with IgAN. Plots of Gd-IgA1 versus Gd-IgA1-specific IgG (A-D) or Gd-IgA1-specific IgA (E-H) in serum samples of patients with IgAN (blue circles; A, E, D, H), CKD controls (red squares; B, F, D, H), or healthy controls (purple triangles; C, G, D, H). Overlays of A-C and E-G are presented in D and H, respectively. Correlation is observed only for Gd-IgA1-specific IgG in IgAN patients (blue circles, r = 0.491) which is depicted by the blue line in panels A and D. No such correlation was observed in either CKD (red) or healthy controls (purple). No correlation was observed for Gd-IgA1-specific IgA autoantibodies. Pearson correlation (r) values and significance (P) are shown for panels A-C and E-G.

A central question in regard to disease progression and therapeutic targeting in IgAN is how the first two hits, Gd-IgA1 and Gd-IgA1-specific IgA and/or IgG autoantibodies, correlate. To address this question, we analyzed the data to see whether there was a correlation between the serum levels of Gd-IgA1 and either Gd-IgA1-specific IgG (Fig 2A–2D) or Gd-IgA1-specific IgA (Fig 2E–2H) in the three cohorts. We observed a significant Pearson correlation between Gd-IgA1 levels and either of the autoantibodies in only the IgAN-patient cohort comparing Gd-IgA1 and Gd-IgA1-specific IgG (Fig 2A) (r = 0.4909, R^2^ = 0.2410, P<0.0001). Similar analysis of the serum levels of Gd-IgA1 and Gd-IgA1-specific IgA in each cohort found no significant correlation between the two biomarkers.

### Composition of autoantibodies

**Fig 3:**
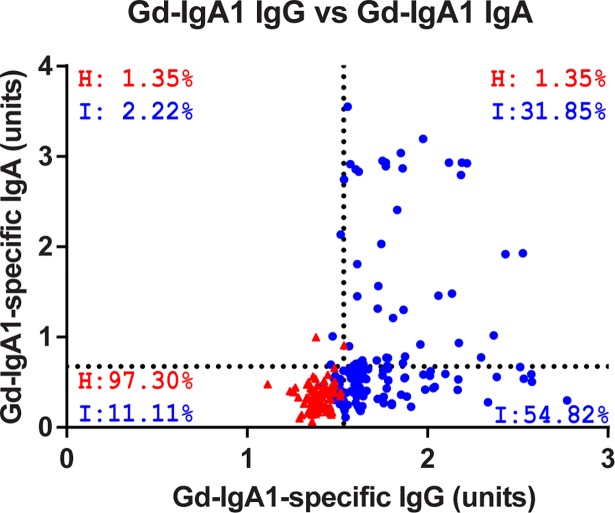
IgAN patients predominantly display increased Gd-IgA1-specific IgG. Plot of the Gd-IgA1-specific IgG versus IgA autoantibodies for the healthy controls (red, H) and IgAN patients (blue, I). Cutoffs were set at 2-standard deviations from the mean values for the healthy controls (1.536 and 0.763 units for IgG and IgA, respectively). Percent occupancy in each quadrant is depicted where occupancy was observed.

Given the overlap in serum levels of Gd-IgA1 in the three cohorts (Fig 1A) and that IgAN patients showed a unique correlation between Gd-IgA1 and Gd-IgA1-specific IgG, we sought to determine whether IgAN patients predominantly exhibited IgG or IgA autoantibodies compared to healthy controls. To determine if individuals with high serum concentrations of Gd-IgA1-specific IgG also had high serum concentrations of Gd-IgA1-specific IgA, or if there was a critical level of total autoantibody that could fully separate the IgAN-patient cohort from the healthy-control cohort, we plotted the relationship between serum levels of Gd-IgA1-specific IgA and Gd-IgA1-specific IgG for the healthy-control and IgAN-patient cohorts (Fig 3). We established four quadrants in the plot, using cut-offs equal to 2 SD from the mean of the healthy-control cohort (1.536 units for Gd-IgA1-specific IgG; 0.763 units for Gd-IgA1-specific IgA). For the IgAN cohort, only 2.2% exhibited high serum levels of Gd-IgA1-specific IgA in the absence of a high level of Gd-IgA1-specific IgG (upper left quadrant), whereas 54.8% of the IgAN patients exhibited increased serum levels of Gd-IgA1-specific IgG without an increased level of Gd-IgA1-specific IgA (lower right quadrant). A smaller portion (31.8%) of the IgAN cohort exhibited increased serum levels of both autoantibodies (upper right quadrant). In total, 86.7% of the IgAN cohort exhibited an increased serum level of Gd-IgA1-specific IgG using the defined cut-offs, whereas only 1.4% of healthy controls were in this group. These data suggest that IgG is the predominant isotype of Gd-IgA1-specific autoantibodies in IgAN.

## Discussion

This study identified a quantitative association between Gd-IgA1 and the corresponding Gd-IgA1-specific IgG autoantibodies which may suggest a causality relationship. Furthermore, the IgG isotype was identified as the predominant autoantibody in IgAN. This study does not provide insight about the population versus individual epitope specificity or the clonality of the Gd-IgA1-specific autoantibody. Gd-IgA1 is a key component of the pathogenic immune complexes in IgAN and its serum level is heritable,[19, 20] due to abnormal expression of key glycosyltransferases in IgA1-producing cells [21–24]. Serum levels of Gd-IgA1 predict disease progression[12] or recurrence after kidney transplantation[25, 26]and multiple studies have attempted to use Gd-IgA1 as a biomarker to discriminate IgAN patients from healthy individuals and patients with other kidney diseases.[17, 27] We found serum Gd-IgA1 levels to be higher in IgAN patients but to have poor discriminative value due to overlap of the data for the disease and healthy controls. Missing in the earlier studies was a correlation between the serum levels of Gd-IgA1 and the corresponding autoantibody, although elevated serum levels of Gd-IgA1 and IgA-IgG immune complexes were observed in IgAN patients with recurrent disease after kidney transplantation.[25] Our data have identified the first direct correlation exclusively in IgAN patients between Gd-IgA1 and Gd-IgA1-specific IgG autoantibodies in this East Asian population. This finding supports the definition of Gd-IgA1 as the key autoantigen in IgAN.

Our multi-hit model led to studies showing that increased serum levels of Gd-IgA1-specific autoantibody predicted disease progression.[11] Yet, determination of the autoantibody isotype was missing. Identification of the isotype is critical in proposing possible therapy due to the differences in the production sites, distribution, and catabolism. Thus, a key finding is our observation that none of the IgAN patients had a high serum level of autoantibody that was exclusively IgA. As a consequence, this study brings into focus the pathological importance of circulatory Gd-IgA1-specific IgG. These autoantibodies share an unusual sequence in the complementarity-determining region 3 of the variable region of their heavy chains that enhances binding to galactose-deficient glycans,[8] apparently as a result of a somatic mutation.[28] The clinical observation that many IgAN patients have renal biopsies without mesangial IgG by standard immunofluorescence staining may seem at odds with our current findings. However, glomerular IgG can be found frequently in these biopsy specimens when using high-resolution confocal microscopy imaging coupled with immunofluorescence staining.[8] Thus, these data together further support the hypothesis that Gd-IgA1-specific IgG likely plays a key role in the pathogenesis of IgAN. Further characterization of this autoantibody as a diagnostic and/or prognostic biomarker in IgAN and for use in monitoring disease progression and responses to therapy is warranted.

## Materials and methods

### Clinical samples and measurement of serum levels of IgA, IgG, Gd-IgA1, and anti-Gd-IgA1 IgG and IgA autoantibodies

Serum samples were previously collected after obtaining written informed consent from 135 patients with biopsy-proven IgAN, 76 patients with other biopsy-proven chronic kidney diseases (CKD), and 106 healthy controls (HC) at Juntendo University, Tokyo, Japan. The institutional review boards at Juntendo University and the University of Alabama at Birmingham approved this study. These cohorts were previously described in detail.[16] S1 Table details the biopsy-proven diagnosis of patients with chronic kidney diseases. Serum levels of IgA, IgG, and Gd-IgA1 were measured by ELISA or lectin-ELISA; anti-Gd-IgA1 IgG and IgA autoantibodies were measured by ELISA using a Fab fragment of Gd-IgA1 as the antigen.[16] IgA and IgG concentrations were expressed as mg/ml; Gd-IgA1, as units/ml; and IgG and IgA autoantibodies, in units relative to IgG or IgA amount.

### Statistical analyses

Statistical analyses and all published plots were prepared using Graphpad Prism 6 software (La Jolla, CA, USA). All Box and Whisker plots depict the 10^th^– 90^th^ percentile for each population with all additional points shown individually. Normality was tested for all populations using the included D’Agostino and Pearson normality test.[18] Comparisons of normally distributed populations were performed using student t-test; non-normally distributed populations were compared using a Mann-Whitney U test. Correlation between samples was assessed using the Pearson correlation coefficient (r).

## Supporting information

S1 Fig Serum concentrations of IgG and IgA and their relationship with their respective autoantibodies or the autoantigen. Box and Whisker plots of 10-90^th^ percentiles with additional points plotted independently for total IgG (A) or IgA (B) in IgAN (circles), CKD (squares), and HC (traingles) serum samples. These values were plotted against their respective Gd-IgA1-specific IgG or IgA autoantibody levels for each cohort in C and D, respectively. We observed no correlation between total immunoglobulin and their respective autoantibodies in any cohort. Total IgG and IgA values were then plotted against Gd-IgA1 autoantigen levels for each cohort in E and F, respectively. No correlation was observed between Gd-IgA1 and total IgG serum levels in any cohort. A shared correlation was observed between Gd-IgA1 and total IgA serum levels across all cohorts (P<0.0001). In C-F, data points are colored as follows: patients with IgAN (blue circles), CKD controls (red squares), or healthy controls (HC, purple triangles). Pearson r values are shown for panel F.
(PDF)

S1 Table Biopsy-proven renal diseases in chronic kidney disease (CKD) controls. (PDF)

## References

[1] Wyatt RJ, Julian BA. IgA nephropathy. N Engl J Med. 2013; 368(25): 2402–14. doi: 10.1056/NEJMra1206793 23782179 10.1056/NEJMra1206793

[2] Berger J, Hinglais N. [Intercapillary deposits of IgA-IgG]. J Urol Nephrol (Paris). 1968; 74(9): 694–5. 4180586

[3] Lai KN, Tang SC, Schena FP, Novak J, Tomino Y, Fogo AB, et al IgA nephropathy. Nat Rev Dis Primers. 2016; 2: 16001 doi: 10.1038/nrdp.2016.1 27189177 10.1038/nrdp.2016.1

[4] Conley ME, Cooper MD, Michael AF. Selective deposition of immunoglobulin A1 in immunoglobulin A nephropathy, anaphylactoid purpura nephritis, and systemic lupus erythematosus. J Clin Invest. 1980; 66(6): 1432–6. doi: 10.1172/JCI109998 6777400 10.1172/JCI109998 PMC371631

[5] Russell MW, Mestecky J, Julian BA, Galla JH. IgA-associated renal diseases: antibodies to environmental antigens in sera and deposition of immunoglobulins and antigens in glomeruli. J Clin Immunol. 1986; 6(1): 74–86. 3514654 10.1007/BF00915367

[6] Suzuki H, Kiryluk K, Novak J, Moldoveanu Z, Herr AB, Renfrow MB, et al The pathophysiology of IgA nephropathy. J Am Soc Nephrol. 2011; 22(10): 1795–803. doi: 10.1681/ASN.2011050464 21949093 10.1681/ASN.2011050464 PMC3892742

[7] Tomana M, Novak J, Julian BA, Matousovic K, Konecny K, Mestecky J. Circulating immune complexes in IgA nephropathy consist of IgA1 with galactose-deficient hinge region and antiglycan antibodies. J Clin Invest. 1999; 104(1): 73–81. doi: 10.1172/JCI5535 10393701 10.1172/JCI5535 PMC408399

[8] Suzuki H, Fan R, Zhang Z, Brown R, Hall S, Julian BA, et al Aberrantly glycosylated IgA1 in IgA nephropathy patients is recognized by IgG antibodies with restricted heterogeneity. J Clin Invest. 2009; 119(6): 1668–77. doi: 10.1172/JCI38468 19478457 10.1172/JCI38468 PMC2689118

[9] Allen AC, Bailey EM, Brenchley PE, Buck KS, Barratt J, Feehally J. Mesangial IgA1 in IgA nephropathy exhibits aberrant O-glycosylation: observations in three patients. Kidney Int. 2001; 60(3): 969–73. doi: 10.1046/j.1523-1755.2001.060003969.x 11532091 10.1046/j.1523-1755.2001.060003969.x

[10] Hiki Y, Odani H, Takahashi M, Yasuda Y, Nishimoto A, Iwase H, et al Mass spectrometry proves under-O-glycosylation of glomerular IgA1 in IgA nephropathy. Kidney Int. 2001; 59(3): 1077–85. doi: 10.1046/j.1523-1755.2001.0590031077.x 11231363 10.1046/j.1523-1755.2001.0590031077.x

[11] Berthoux F, Suzuki H, Thibaudin L, Yanagawa H, Maillard N, Mariat C, et al Autoantibodies targeting galactose-deficient IgA1 associate with progression of IgA nephropathy. J Am Soc Nephrol. 2012; 23(9): 1579–87. doi: 10.1681/ASN.2012010053 22904352 10.1681/ASN.2012010053 PMC3431415

[12] Zhao N, Hou P, Lv J, Moldoveanu Z, Li Y, Kiryluk K, et al The level of galactose-deficient IgA1 in the sera of patients with IgA nephropathy is associated with disease progression. Kidney Int. 2012; 82(7): 790–6. doi: 10.1038/ki.2012.197 22673888 10.1038/ki.2012.197 PMC3443545

[13] Bellur SS, Troyanov S, Cook HT, Roberts ISD on behalf of a Working Group of International IgA Nephropathy Network and Renal Pathology Society. Immunostaining findings in IgA nephropathy: correlation with histology and clinical outcome in the Oxford classification patient cohort. Nephrol Dial Transplant. 2011; 26(8): 2533–6. doi: 10.1093/ndt/gfq812 21273233 10.1093/ndt/gfq812

[14] Shin DH, Lim BJ, Han IM, Han SG, Kwon YE, Park KS, et al Glomerular IgG deposition predicts renal outcome in patients with IgA nephropathy. Mod Pathol. 2016; 29(7): 743–52. doi: 10.1038/modpathol.2016.77 27102346 10.1038/modpathol.2016.77

[15] Arroyo AH, Bomback AS, Butler B, Radhakrishnan J, Herlitz L, Stokes MB, et al Predictors of outcome for severe IgA Nephropathy in a multi-ethnic U.S. cohort. Clin Nephrol. 2015; 84(3): 145–55. doi: 10.5414/CN108556 26226949 10.5414/CN108556

[16] Yanagawa H, Suzuki H, Suzuki Y, Kiryluk K, Gharavi AG, Matsuoka K, et al A panel of serum biomarkers differentiates IgA nephropathy from other renal diseases. PLoS One. 2014; 9(5): e98081 doi: 10.1371/journal.pone.0098081 24858067 10.1371/journal.pone.0098081 PMC4032235

[17] Moldoveanu Z, Wyatt RJ, Lee JY, Tomana M, Julian BA, Mestecky J, et al Patients with IgA nephropathy have increased serum galactose-deficient IgA1 levels. Kidney Int. 2007; 71(11): 1148–54. doi: 10.1038/sj.ki.5002185 17342176 10.1038/sj.ki.5002185

[18] D'Agostino RB. Transformation to normality of the null distibution of g1. Biometrika. 1970; 57(3): 679–81.

[19] Gharavi AG, Moldoveanu Z, Wyatt RJ, Barker CV, Woodford SY, Lifton RP, et al Aberrant IgA1 glycosylation is inherited in familial and sporadic IgA nephropathy. J Am Soc Nephrol. 2008; 19(5): 1008–14. doi: 10.1681/ASN.2007091052 18272841 10.1681/ASN.2007091052 PMC2386728

[20] Hastings MC, Moldoveanu Z, Julian BA, Novak J, Sanders JT, McGlothan KR, et al Galactose-deficient IgA1 in African Americans with IgA nephropathy: serum levels and heritability. Clin J Am Soc Nephrol. 2010; 5(11): 2069–74. doi: 10.2215/CJN.03270410 20634323 10.2215/CJN.03270410 PMC3001782

[21] Kiryluk K, Li Y, Moldoveanu Z, Suzuki H, Reily C, Hou P, et al GWAS for serum galactose-deficient IgA1 implicates critical genes of the *O*-glycosylation pathway. PLoS Genet. 2017; 13(2): e1006609 doi: 10.1371/journal.pgen.1006609 28187132 10.1371/journal.pgen.1006609 PMC5328405

[22] Suzuki H, Moldoveanu Z, Hall S, Brown R, Vu HL, Novak L, et al IgA1-secreting cell lines from patients with IgA nephropathy produce aberrantly glycosylated IgA1. J Clin Invest. 2008; 118(2): 629–39. doi: 10.1172/JCI33189 18172551 10.1172/JCI33189 PMC2157566

[23] Suzuki H, Raska M, Yamada K, Moldoveanu Z, Julian BA, Wyatt RJ, et al Cytokines alter IgA1 O-glycosylation by dysregulating C1GalT1 and ST6GalNAc-II enzymes. J Biol Chem. 2014; 289(8): 5330–9. doi: 10.1074/jbc.M113.512277 24398680 10.1074/jbc.M113.512277 PMC3931088

[24] Gale DP, Molyneux K, Wimbury D, Higgins P, Levine AP, Caplin B, et al Galactosylation of IgA1 Is associated with common variation in C1GALT1. J Am Soc Nephrol. 2017; 28(7): 2158–66. doi: 10.1681/ASN.2016091043 28209808 10.1681/ASN.2016091043 PMC5491291

[25] Berthelot L, Robert T, Vuiblet V, Tabary T, Braconnier A, Drame M, et al Recurrent IgA nephropathy is predicted by altered glycosylated IgA, autoantibodies and soluble CD89 complexes. Kidney Int. 2015; 88(4): 815–22. doi: 10.1038/ki.2015.158 26061544 10.1038/ki.2015.158

[26] Berthoux F, Suzuki H, Mohey H, Maillard N, Mariat C, Novak J, et al Prognostic value of serum biomarkers of autoimmunity for recurrence of IgA nephropathy after kidney transplantation. J Am Soc Nephrol. 2017; 28(6): 1943–50. doi: 10.1681/ASN.2016060670 28255003 10.1681/ASN.2016060670 PMC5461789

[27] Suzuki Y, Matsuzaki K, Suzuki H, Okazaki K, Yanagawa H, Ieiri N, et al Serum levels of galactose-deficient immunoglobulin (Ig) A1 and related immune complex are associated with disease activity of IgA nephropathy. Clin Exp Nephrol. 2014; 18(5): 770–7. doi: 10.1007/s10157-013-0921-6 24477513 10.1007/s10157-013-0921-6 PMC4194014

[28] Huang ZQ, Raska M, Stewart TJ, Reily C, King RG, Crossman DK, et al Somatic mutations modulate autoantibodies against galactose-deficient IgA1 in IgA nephropathy. J Am Soc Nephrol. 2016; 27(11): 3278–84. doi: 10.1681/ASN.2014101044 26966014 10.1681/ASN.2014101044 PMC5084875

