# Supplementary material for: Serum galactose-deficient-IgA1 and IgG autoantibodies correlate in patients with IgA nephropathy
Source: PLoS One. 2018 Jan 11;13(1):e0190967. doi: 10.1371/journal.pone.0190967 (PMC5764330; doi:10.1371/journal.pone.0190967)
Supplement: S1 Fig — Box and Whisker plots of 10-90th percentiles with additional points plotted independently for total IgG (A) or IgA (B) in IgAN (circles), CKD (squares), and HC (traingles) serum samples. These values were plotted against their respective Gd-IgA1-specific IgG or IgA autoantibody levels for each cohort in C and D, respectively. We observed no correlation between total immunoglobulin and their respective autoantibodies in any cohort. Total IgG and IgA values were then plotted against Gd-IgA1 autoantigen levels for each cohort in E and F, respectively. No correlation was observed between Gd-IgA1 and total IgG serum levels in any cohort. A shared correlation was observed between Gd-IgA1 and total IgA serum levels across all cohorts (P<0.0001). In C-F, data points are colored as follows: patients with IgAN (blue circles), CKD controls (red squares), or healthy controls (HC, purple triangles). Pearson r values are shown for panel F. (PDF) [file pone.0190967.s001.pdf]

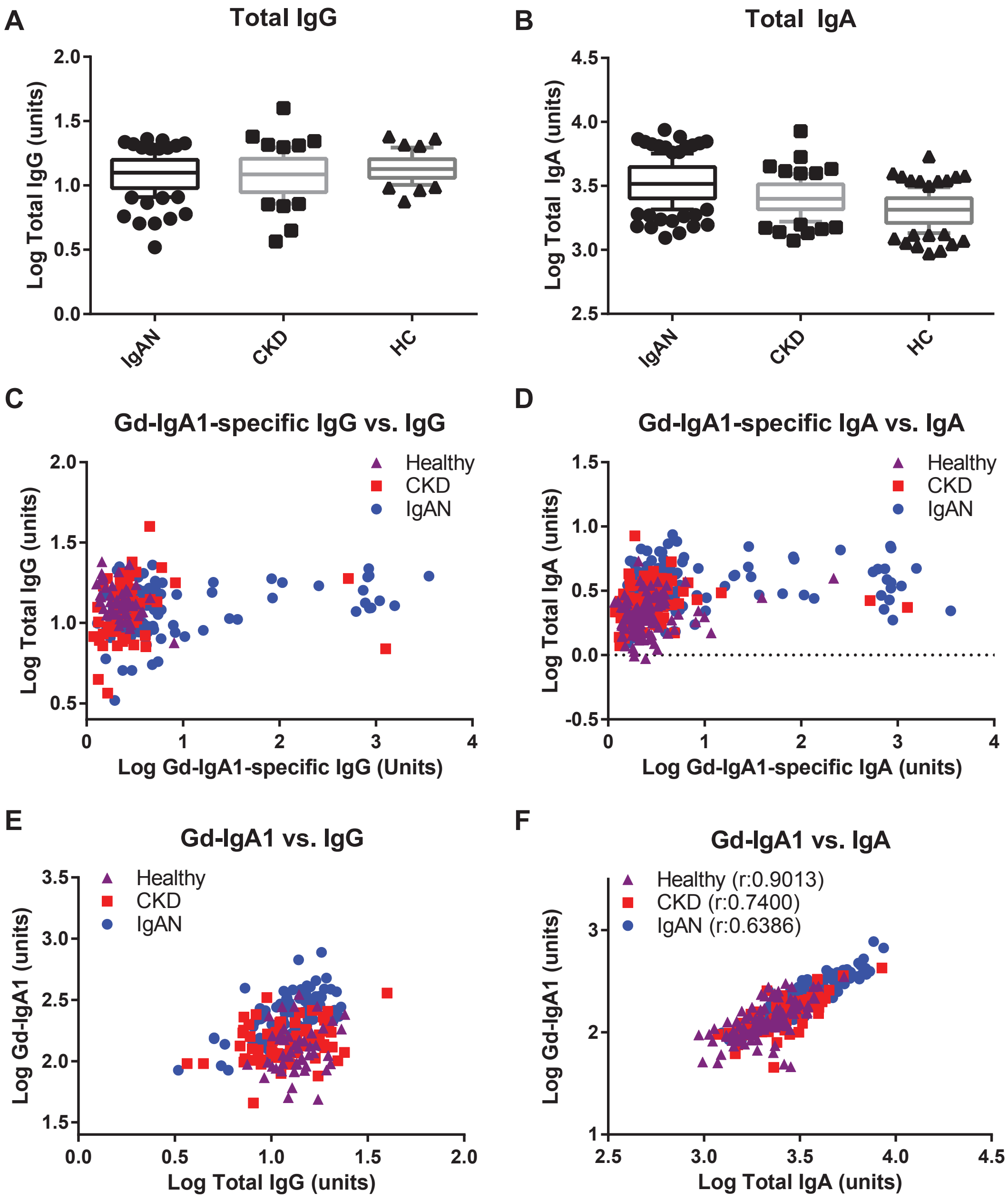

Supplementary S1 Figure. Serum concentrations of IgG and IgA and their relationship with their respective autoantibodies or the autoantigen. Box and Whisker plots of 10-90th percentiles with additional points plotted independently for total IgG (A) or IgA (B) in IgAN (circles), CKD (squares), and HC (triangles) serum samples. These values were plotted against their respective Gd-IgA1-specific IgG or IgA autoantibody levels for each cohort in C and D, respectively. We observed no correlation between total immunoglobulin and their respective autoantibodies in any cohort. Total IgG and IgA values were then plotted against Gd-IgA1 autoantigen levels for each cohort in E and F, respectively. No correlation was observed between Gd-IgA1 and total IgG serum levels in any cohort. A shared correlation was observed between Gd-IgA1 and total IgA serum levels across all cohorts (P<0.0001). In C-F, data points are colored as follows: patients with IgAN (blue circles), CKD controls (red squares), or healthy controls (HC, purple triangles). Pearson r values are shown for panel F.
