# Supplementary material for: Serum galactose-deficient-IgA1 and IgG autoantibodies correlate in patients with IgA nephropathy
Source: PLoS One. 2018 Jan 11;13(1):e0190967. doi: 10.1371/journal.pone.0190967 (PMC5764330; doi:10.1371/journal.pone.0190967)
Supplement: S1 Table — (PDF) [file pone.0190967.s002.pdf]

Supplementary S1 Table

Biopsy-proven renal diseases in chronic kidney disease (CKD) controls.

Renal disease	N
Diabetic nephropathy	30
Membranous nephropathy	9
Non-IgAN proliferative nephropathy	9
Lupus nephritis	8
Minimal change nephrotic syndrome	6
Nephrosclerosis	4
Interstitial nephritis	2
Focal segmental glomerulosclerosis	2
ANCA-related nephritis*	1
Scleroderma renal crisis	1
HCV-related nephritis+	1
Fabry's disease	1
Acute post-streptococcal nephritis	1
Minor glomerular abnormality	1

*ANCA: anti-neutrophil cytoplasmic antibody

+HCV: hepatitis C virus
